# Conserved Amphipathic Helices Mediate Lipid Droplet Targeting of Perilipins 1–3*

**DOI:** 10.1074/jbc.M115.691048

**Published:** 2016-01-07

**Authors:** Emily R. Rowe, Michael L. Mimmack, Antonio D. Barbosa, Afreen Haider, Iona Isaac, Myriam M. Ouberai, Abdou Rachid Thiam, Satish Patel, Vladimir Saudek, Symeon Siniossoglou, David B. Savage

**Affiliations:** From the ‡ University of Cambridge Metabolic Research Laboratories, Wellcome Trust-Medical Research Council Institute of Metabolic Science, Cambridge CB2 0QQ, United Kingdom,; the § Cambridge Institute for Medical Research, University of Cambridge, Cambridge CB2 0XY, United Kingdom,; the ¶ Nanoscience Centre, Department of Engineering, University of Cambridge, Cambridge CB3 0FF, United Kingdom, and; the ‖ Laboratoire de Physique Statistique, Ecole Normale Supérieure de Paris, Université Pierre et Marie Curie, Université Paris Diderot, CNRS, 24 Rue Lhomond, 75005 Paris, France

**Keywords:** lipid droplet, lipodystrophy, lipolysis, membrane, phospholipid, 11-mer repeat, amphipathic helix, membrane targeting, monolayer, perilipin

## Abstract

Perilipins (PLINs) play a key role in energy storage by orchestrating the activity of lipases on the surface of lipid droplets. Failure of this activity results in severe metabolic disease in humans. Unlike all other lipid droplet-associated proteins, PLINs localize almost exclusively to the phospholipid monolayer surrounding the droplet. To understand how they sense and associate with the unique topology of the droplet surface, we studied the localization of human PLINs in *Saccharomyces cerevisiae,* demonstrating that the targeting mechanism is highly conserved and that 11-mer repeat regions are sufficient for droplet targeting. Mutations designed to disrupt folding of this region into amphipathic helices (AHs) significantly decreased lipid droplet targeting *in vivo* and *in vitro*. Finally, we demonstrated a substantial increase in the helicity of this region in the presence of detergent micelles, which was prevented by an AH-disrupting missense mutation. We conclude that highly conserved 11-mer repeat regions of PLINs target lipid droplets by folding into AHs on the droplet surface, thus enabling PLINs to regulate the interface between the hydrophobic lipid core and its surrounding hydrophilic environment.

## Introduction

Triacylglycerol storage within LDs^2^ enables individual cells to limit the toxic effects of non-esterified fatty acid accumulation and higher order metazoans to store surplus energy for subsequent use during exercise or fasting. A surface phospholipid monolayer provides an amphipathic interface between the neutral lipid core and surrounding hydrophilic cytoplasm (1, 2). Lipases catalyze the sequential hydrolysis of triacylglycerol, enabling cells to balance lipid storage with subsequent lipid breakdown for oxidation, metabolism or, in the case of adipocytes, systemic release into the circulation (3, 4). Adult humans, even in the lean state, store >90% (600–800 MJ) of their surplus energy within adipocyte LDs, so regulating when and to what extent these reserves are released is critical to good health (5).

Given this need to precisely coordinate intracellular lipolysis, it is not surprising that lipase activity is subject to several “regulatory steps” (3, 4). Among these, PLINs, a family of five proteins, have been shown to orchestrate both triacylglycerol and diacylglycerol lipase activity, potentially even channeling substrates between these enzymes (6–10). The particular importance of PLIN1 in regulating lipolysis in adipocytes was recently highlighted by the severe metabolic phenotype of patients with loss-of-function mutations in *PLIN1* (11, 12). The *PLIN1* mutations impair suppression of basal lipolysis and co-segregate with partial lipodystrophy, severe insulin resistance, type 2 diabetes, dyslipidemia, and fatty liver disease, emphasizing the importance of finely tuned intracellular lipolytic regulation in adipocytes. In the basal or non-stimulated state, the carboxyl terminus of PLIN1 binds α/β-hydrolase fold domain containing protein 5 (ABHD5), an adipose triglyceride lipase (ATGL) co-activator, stabilizing its expression (13, 14) and sequestering it from ATGL (7). Protein kinase A (PKA) activation, triggered by lipolytic stimuli, results in phosphorylation of several PLIN1 sites and leads to the release of ABHD5, which then binds and activates ATGL (7), dramatically increasing its hydrolytic activity (15). PKA-induced phosphorylation of PLIN1 coincides with PKA-mediated phosphorylation of hormone-sensitive lipase (HSL), the major diacylglycerol lipase, facilitating binding to PLIN1 on the LD surface and enzyme activation (6, 8, 16). These pathways have been most clearly elucidated for PLIN1, but other PLINs also interact with HSL (17) and ABHD5 (18–20).

Clearly their position on the surface of LDs is a critical element in enabling PLINs to regulate lipolysis, so it is not surprising that this targeting is evolutionarily conserved in all cells types in which PLINs are present. PLIN1 is the principal adipocyte LD coat protein, but other PLINs are present on the surface of LDs in almost all eukaryotic cell types (6). When not associated with this interface, PLINs 1 and 2 are rapidly degraded (21–25), whereas PLIN3 is also stable in the cytoplasm where it may have additional trafficking roles (26–29). Whereas other proteins, characterized by having a monotopic (meaning that they only span one of the two phospholipid layers) membrane spanning region, move between the endoplasmic reticulum and LD surface (30–32), PLINs are thought to be synthesized on cytosolic ribosomes from which they directly target LDs (21, 33). How then do they locate this specific intracellular site?

Several deletion studies (34–45) have attempted to define the targeting determinants of PLINs 1–3, but consensus has yet to emerge (6). The only available structural data related to PLINs comes from the solution (46) and crystal (47) structure of the carboxyl terminus of PLIN3, which was shown to be able to fold into a 4-helix bundle stabilized by an α/β-domain zipped together by two β-sheets (47). Homology analysis suggests that a similar helix bundle is likely to be present in PLINs 2 and 5 and possibly also PLIN1 (6, 42, 47, 48). This motif could, given the necessary energetic trigger, unfold and then associate with a membrane by virtue of the otherwise concealed hydrophobic faces of the helices. Alternatively, sequences with a characteristic 11-mer repeat pattern are present in all PLINs, and they too could conceivably be involved in LD targeting (44, 49).

We were prompted to review this question for several reasons. First, mechanistic understanding of LD targeting by proteins remains limited (50, 51). Second, appropriate targeting of PLINs, the most abundant LD coat proteins, to this interface is clearly critical to their function in optimizing energy storage and release. Third, while characterizing the mutant forms of PLIN1 that we recently identified, we noted that all three mutants retain LD targeting capacity (11, 13). Furthermore, we hypothesize that LD targeting of the mutant proteins is a “necessary” element in their pathogenicity as it results, we assume, in the presence of both WT and mutant PLIN1 on the surface of LDs, whereas we hypothesize that heterozygous mutants, which do not target LDs, would not have a dominant and therefore overt clinical phenotype. Furthermore, as some of these mutants would be predicted to impact upon the putative 4-helix bundle of PLIN1, these findings arguably favor the amino-terminal 11-mer repeat regions as primary LD targeting determinants.

## Experimental Procedures

### 

#### 

##### Cloning Strategies

Human *PLIN1, -2,* and -*3* cDNA was cloned in-frame into YCplac111-NOP-GFP (*CEN, LEU2,* and *NOP1* promoter) at BamHI or NotI restriction sites to generate either amino- or carboxyl-terminal GFP-tagged constructs, respectively. To study AH membrane targeting, the *PLIN1* 11-mer repeat region (amino acids 93–192) was cloned in-frame into pCLJ730 (2 μm, *URA3*, TetO2-GMAP210 amino acids 39–377-yEGFP, obtained from Dr. C. Jackson) between the BamHI and AgeI restriction sites, resulting in a carboxyl-terminal coiled-coil (CC) GFP tag (52). The synapsin I AH (amino acids 69–96) was provided as a non-LD targeting control. For expression in mammalian cells, each AH-CC-GFP sequence was amplified and subcloned into pcDNA 3.1. The QuikChange II XL site-directed mutagenesis kit (Agilent Technologies, Santa Clara, CA) was used to generate disruptive and conservative mutations, which were chosen based on helical wheels generated using Heliquest (53). Following mutagenesis, mutated sequences were subcloned into the original YCplac111-NOP-GFP or pCLJ730 vector.

For purification of the PLIN1 11-mer repeat region (amino acids 93–192), WT and mutant (L143D) DNA fragments were subcloned into pET22-CPD_SalI_ (54). The mutation C150A was incorporated into each clone to eliminate non-native disulfide bridge formation during purification. All plasmids generated in this study were confirmed by DNA sequencing.

##### Yeast Strains, Media, and Growth Conditions

The yeast strains used in this study are recorded in Table 1. Yeast cells were grown aerobically at 30 °C in a gyrator shaker at 210 rpm. Cells were transformed with the above plasmids and grown in synthetic complete drop-out medium consisting of 0.2% (w/v) yeast nitrogen base (BD Biosciences), 0.6% (w/v) ammonium sulfate (Fisher Scientific, Loughborough, UK), 2% glucose, and appropriate amino acids (Sigma); for selection of YCplac111 and pCLJ730-derived plasmids, yeast cells were grown in media lacking leucine and uracil, respectively.

**TABLE 1:** ***S. cerevisiae* strains used in this study**

Strain	Genotype	Ref./source
RS453 (WT strain)	*MAT*α *ade2-1 his3-11,15 ura3–52 leu2-3,112 trp1-1*	81
*ERG6-*mCherry	*ERG6-mCherry*::*KanMX6*, derived from RS453	61

##### Confocal Microscopy of Saccharomyces cerevisiae

Yeast cells were grown to either logarithmic (*A*_600_ = ∼0.6–0.8) or post-diauxic shift (*A*_600_ >3.5) phase, pelleted, and resuspended in a small volume of media. 2.5 μl of this suspension was mounted on a glass slide and covered with a glass coverslip. Images were acquired at room temperature on a Leica TCS SP8 (Leica, Wetzlar, Germany) confocal microscope using the white light laser and a 63 × 1.4NA oil objective at zoom 4. Logarithmic phase cells and post-diauxic shift phase cells were imaged using the standard and resonant scanner (8000 Hz scanning speed), respectively. Sequential scanning was always used. Where relevant, logarithmic cells were incubated with 0.2 μm MitoTracker Orange CMTMRos (Life Technologies, Inc.) for 30 min and washed in fresh media before preparation for microscopy. The following excitation/emissions were used: GFP (488 nm/494–562 nm); mCherry (587 nm/595–719 nm); MitoTracker Orange (551 nm/570–620 nm); GFP when MitoTracker was used (488 nm/499–550 nm). Microscopy settings were kept constant within an experiment if signal intensity was to be compared. To aid visualization, the brightness and contrast of images was adjusted using Photoshop CS6 (Adobe, San Jose, CA). Only raw data were used for quantification.

##### Quantification of Protein Localized to Erg6-mCherry Labeled Lipid Droplets

Image processing was performed using Volocity 6.3 software (PerkinElmer Life Sciences). Objects larger than 0.05 μm^2^ were identified in the mCherry (population 1) and GFP (population 2) channels. The intersection of the populations was calculated and identified as GFP and Erg6-mCherry-labeled LDs (population 3). To quantify the amount of GFP localized to Erg6-mCherry-labeled LDs, the number of pixels in the GFP channel of population 3 was divided by the area of population 1 or the total population 2 (as indicated) and calculated as a percentage of the mean of the WT from the same experiment. Quantification settings were kept the same within a single experiment.

##### COS-7 Culture and Transfection

COS-7 cells (obtained from ATCC, Manassas, VA) were cultured in DMEM (Sigma) supplemented with 10% (v/v) fetal bovine serum (HyClone, South Logan, UT). Cells were seeded in 12-well plates at a density of 40,000 cells/well and transfected the following day with 500 ng of DNA/well using Lipofectamine LTX with Plus reagent (Life Technologies, Inc.). Oleic acid/albumin (400 μm; Sigma) was added to cells 5–6 h post-transfection.

##### Confocal Microscopy of COS-7 Cells

48 h post-transfection, cells grown on coverslips were fixed in 4% (v/v) formaldehyde solution, and LDs were stained with 0.1% LipidTOX Deep Red Neutral Lipid Stain (Life Technologies, Inc.) prior to mounting in ProLong Gold antifade mounting reagent with DAPI (Life Technologies, Inc.). Images were acquired on a Leica TCS SP8 confocal microscope using the 405 nm diode and white light lasers and a 63 × 1.4NA oil objective. Sequential scanning was used. The following excitation/emissions were used: GFP (488 nm/495–561 nm), LipidTOX (633/641–700 nm), and DAPI (405 nm/415–470 nm).

##### Cycloheximide Chase

Logarithmic ERG6-mCherry cells were split into 10-ml fractions for cell recovery at different time points. One fraction was reserved as a measure of the starting point of the chase (0 min). Cycloheximide (50 μg/ml) was added to the remaining fractions, and cultures were further incubated at 30 °C. At the indicated time points, the *A*_600_ was recorded to normalize protein loading for immunoblotting, and cells were pelleted and processed for immunoblotting as described below. Bands were quantified using Image Lab Software (Bio-Rad), and the GFP band intensity was normalized to that of GAPDH.

##### Immunoblotting

Logarithmic yeast cells (∼4 *A*_600_) were pelleted and washed with water. Pelleted cells were lysed with glass beads (Thistle Scientific, Glasgow, UK) in NuPAGE LDS sample buffer (LDS; Life Technologies, Inc.) containing 2.5% (v/v) β-mercaptoethanol (Sigma) by two rounds of vortexing (30s)/boiling (2 min) followed by a final 2-min vortex. The volume of LDS added was dependent on the *A*_600_ of the sample. Extracts were centrifuged at 20,000 × *g* for 15 min, and the supernatant was resolved by SDS-PAGE.

COS-7 cells were washed twice in ice-cold PBS, and protein was harvested in RIPA buffer (Sigma) containing a protease inhibitor mixture (Roche Applied Science, Basel, Switzerland) and phosSTOP (Roche Applied Science). Extracts were centrifuged at 16,100 × *g* for 15 min at 4 °C, and the supernatant protein concentration was determined using the DC protein assay (Bio-Rad). Proteins were denatured in LDS at 95 °C for 5 min.

Yeast and COS-7 lysate prepared in LDS were resolved by SDS-PAGE, and protein was transferred to nitrocellulose membrane using the iBLOT apparatus (Life Technologies, Inc.). Membranes were blocked for 1 h in 5% (w/v) dried skimmed milk (Marvel), and membranes were probed overnight at 4 °C with the following primary antibodies: rabbit anti-GFP (1:1000 dilution in 5% milk; Abcam catalog no. ab290, lot no. GR135929-1 and GR158277-1); mouse anti-GFP (1:1000 in 3% BSA; Roche Applied Science catalog no. 11814460001, lot no. 11063100); and mouse anti-GAPDH (1:1000 in 5% milk; Genetex catalog no. GT239, lot no. 41323). Membranes were washed and incubated for 1 h with either HRP-conjugated anti-rabbit IgG or HRP-conjugated anti-mouse IgG, followed by a final washing step. Blots were visualized using Amersham Biosciences ECL (GE Healthcare, UK) or Immobilon Western chemiluminescent HRP substrate (Millipore, Darmstadt, Germany).

##### RNA Extraction from S. cerevisiae and Quantitative RT-PCR Analysis

Logarithmic yeast cells (∼6–8 *A*_600_) were pelleted, washed with water, and snap-frozen in liquid nitrogen. Cells were lysed by mechanical disruption in buffer RLT (Qiagen, Venlo, Netherlands) and lysing matrix C (MP Bio, Santa Ana, CA) using the Fast Prep 24 homogenizer (MP Bio). The lysate was centrifuged at 20,000 × *g,* and total RNA in the supernatant was purified using the RNeasy mini kit (Qiagen). Total RNA was used to generate cDNA using Superscript II reverse transcriptase (Life Technologies, Inc.). mRNA expression of *PLIN1*-(1–192) and housekeeping genes (*ACT1* and *PDA1*) were measured by real time quantitative RT-PCR using either TaqMan MasterMix (ABI, Applied Biosystems Inc, Foster City, CA) or SYBR Green Mastermix (ABI), respectively, on an ABI 7300 RT-PCR system. The gene expression assays and primer sequences used are listed in Table 2. For each sample the expression of *PLIN1*-(1–192) mRNA was normalized to the geomean of the two housekeeping genes and calculated as a percentage of the mean of the WT samples.

**TABLE 2:** **Real time quantitative RT-PCR gene expression assays and primer sequences**

Gene	Gene expression assay/primer sequences
*PLIN1*	Hs00160173_m1 (ABI)
*ACT1*	Forward, TGGATTCTGAGGTTGCTGCT
	Reverse, GGTGTCTTGGTCTACCGACG
*PDA1*	Forward, AGAGGTGCCTCAGTGAAAGC
	Reverse, GAAGCCTGGAGCGTAAAGGT

##### Expression and Purification of PLIN1 11-mer Repeat Region

Plasmids were transformed into the expression strain NiCo21 (New England Biolabs, Hitchin, UK). Overnight cultures were diluted into 1 liter of lysogeny broth supplemented with 0.2% glucose and grown with shaking at 37 °C to *A*_600_ = 1.0. 1 mm isopropyl β-d-1-thiogalactopyranoside was added, and cultures were further grown for 80 min at 37 °C. Cell pellets from a 500-ml culture were resuspended in ∼14 ml of B-PER (Thermo Scientific) with added 10% glycerol, 4 mg of lysozyme (Sigma), 500 units of Pierce Universal Nuclease (Thermo Scientific), and complete EDTA-free protease inhibitors (Roche Applied Science). Cell lysates were rotated at room temperature for 15 min, centrifuged at 28,000 × *g* for 30 min, and filtered (Minisart 0.22 μm; Sartorius, Göttingen, Germany). Affinity purification was performed using 0.5–1.0 ml of nickel-nitrilotriacetic acid-agarose beads (Qiagen) while rotating for 1 h at 4 °C, before bound fusion proteins were transferred to the CPD reaction buffer (20 mm Tris, 60 mm NaCl, 250 mm sucrose, 3 mm imidazole, pH 7.4). Cleaved products were eluted into the supernatant using 50–100 μm inositol hexakisphosphate during rotation for 2 h at 4 °C (54). Isolation of purified proteins was performed using an AKTA pure chromatography system and Superdex^TM^ 75 10/300 GL column (GE Healthcare). Protein concentration for lipid droplet flotation assay and circular dichroism (CD) was determined by amino acid analysis (55). To confirm purity, equal amounts of protein were resolved on an 18% Tris-glycine gel (Life Technologies, Inc.) and stained with quick Coomassie stain (Generon, Maidenhead, UK).

##### Lipid Droplet Flotation Assay

1,2-Dioleoyl-*sn*-glycero-3-phosphocholine (0.617 mg) and 1,2-dioleoyl-*sn*-glycero-3-phosphoethanolamine (0.38 mg) prepared in chloroform (Avanti Lipids, Alabaster, AL) were mixed and dried under a stream of nitrogen. Phospholipids were solubilized in 200 μl of glyceryl trioleate (Sigma) by vigorous vortexing. 100 μl of solubilized lipid was diluted 10× in 25 mm Tris, pH 7.4, 1 mm MgCl_2_ (LD buffer) and mixed by vortexing and sonication, resulting in a white LD emulsion. LDs were further diluted two times and stored on ice until use. LDs were vortexed immediately prior to use and were used on the same day as preparation. The protocol for the LD flotation assay was based on that described previously in two studies (56, 57). Artificial LDs and protein (0.5–1.0 μm) were mixed in a total volume of 210 μl and rotated at room temperature for 30 min. The sample was adjusted to 30% (w/v) sucrose by adding 140 μl of 75% (w/v) sucrose prepared in LD buffer, and 320–330 μl layered at the bottom of a 0.8-ml ultra-clear tube (Beckman Coulter, High Wycombe, UK). The sample was overlaid with 260 μl of 25% (w/v) sucrose prepared in LD buffer and finally 50–60 μl of LD buffer. The sample was centrifuged at 25,000 rpm (76,000 × *g*) in an SW55 Ti rotor (Beckman Coulter) at 20 °C for 3 h. The top (100 μl), middle (260 μl), and remaining bottom fractions were collected and frozen prior to analysis by SDS-PAGE. The parameters used were kept the same within a single experiment. Equal volumes of each fraction were prepared in LDS buffer and resolved on an 18% Tris-glycine gel (Life Technologies. Inc.). Silver staining was performed using the SilverQuest Silver Staining Kit (Life Technologies, Inc.). Band intensities were quantified using ImageLab software, and bound protein was calculated as the amount of protein in the top fraction divided by the sum of protein in the top and bottom fractions.

##### Circular Dichroism

CD spectra were recorded on an Aviv Model 410 (Aviv Biomedical Inc., Lakewood, NJ) at 25 °C using a 0.1-cm quartz cuvette. Protein was prepared in either 10 mm potassium phosphate buffer, pH 7.4, or 25 mm Tris, pH 7.4, 1 mm MgCl_2_ with or without 0.05% (v/v) *N*,*N*-dimethyldodecylamine *N*-oxide (LDAO; Sigma) at concentrations ranging from 2.6 to 10 μm. Spectra were recorded between 190 and 260 nm in increments of 1 nm, with a bandwidth of 1 nm and an averaging time of 5 s. Spectra from three scans were averaged, baseline corrected, and smoothed using the Aviv software. Spectra are expressed as mean residue ellipticity using protein concentrations determined by amino acid analysis. CD spectra were deconvoluted with the CDSSTR algorithm using the Dichroweb software (58, 59). The estimated helical content given was obtained when using protein reference set 4 (60). Normalized mean root square deviation was <0.011 for all deconvolutions described.

##### Statistics

Quantitative data are expressed as mean ± S.D. Multiple group comparisons were determined with one-way ANOVA with Dunnett's multiple comparisons test using GraphPad Prism software (GraphPad, San Diego). Comparisons between two independent groups were assessed using unpaired Student's *t* tests. Statistical significance was defined as *p* < 0.05.

## Results

PLINs are present in essentially all mammalian cell types and “compete” for LD association, meaning that it is very difficult to generate a PLIN null mammalian cell line in which one could study the localization of tagged PLINs without the latter being influenced by endogenous PLIN. Therefore, to address this question, we elected to use *S. cerevisiae* as it is not known to have a PLIN orthologue.

**FIGURE 1.**
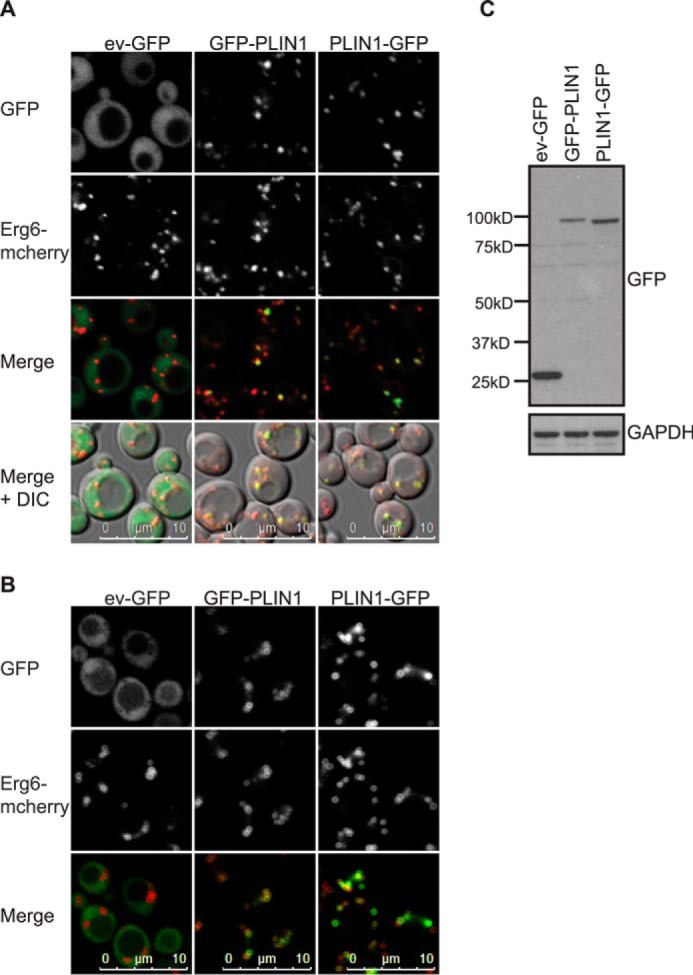
**Human PLIN1 localizes to Erg6-mCherry-labeled lipid droplets in *S. cerevisiae*.** *A* and *B,* ERG6-mCherry cells expressing PLIN1 tagged at either the amino or carboxyl terminus were grown in selective media to log (*A*) or post-diauxic shift (*B*) phase and imaged by confocal microscopy. The brightness and contrast of images were adjusted to aid visualization. Both GFP-tagged PLIN1 proteins localized to Erg6-mCherry-labeled LDs. Empty vector (*ev*) GFP was cytosolic. Images shown are representative of multiple images acquired from three (*A*) or two (*B*) separate experiments. *C,* GFP immunoblotting of cells described in *A*. GAPDH was used as a loading control. *DIC*, differential interference contrast.

**FIGURE 2.**
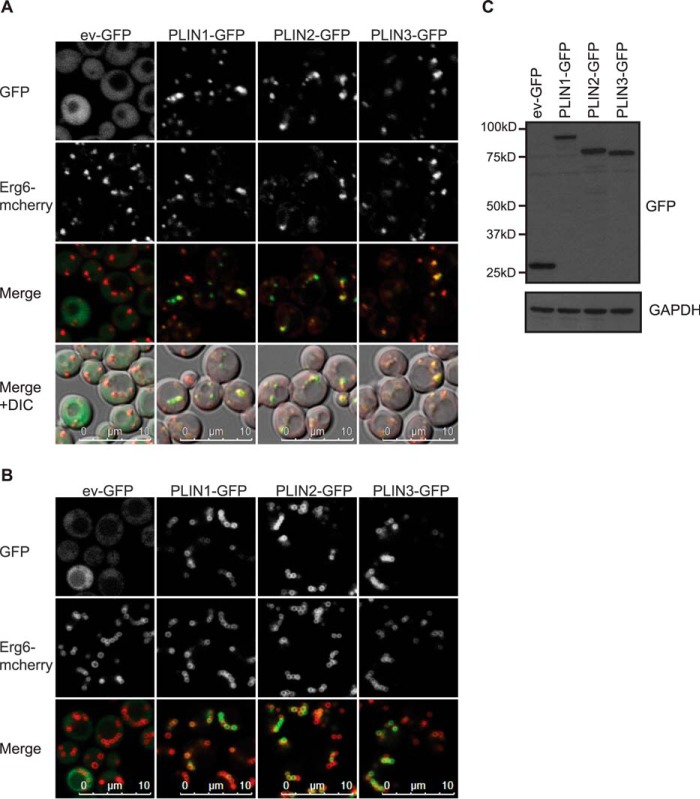
**Human PLINs 1–3 localize to Erg6-mCherry-labeled lipid droplets in *S. cerevisiae*.** *A* and *B,* ERG6-mCherry cells expressing carboxyl-terminal GFP tagged PLINs 1–3 were grown in selective media to log (*A*) or post-diauxic shift (*B*) phase and imaged by confocal microscopy. The brightness and contrast of images were adjusted to aid visualization. PLINs 1–3 all co-localized with Erg6-mCherry-labeled LDs in both growth phases. Images shown are representative of multiple images acquired in three (*A*) or two (*B*) separate experiments. *C,* GFP immunoblot of lysates from the cells described in *A*. GAPDH was used as a loading control. *DIC*, differential interference contrast; *ev,* empty vector.

We began by confirming that, as reported previously (61), mCherry-tagged Erg6 expressed from the endogenous locus (ERG6-mCherry cells) co-localizes with LDs stained with a neutral lipid dye (data not shown). We then expressed human GFP-tagged PLINs 1–3 in ERG6-mCherry cells. Regardless of which terminus of PLIN1 was tagged with GFP, LD localization was confirmed (Fig. 1). Similarly, GFP-tagged human PLINs 2 and 3 targeted LDs in these cells (Fig. 2). In itself, this observation is informative as it suggests that the targeting mechanism is highly conserved and, given the vast evolutionary distance between yeast and humans, is unlikely to involve direct interactions with other yeast proteins. This finding has also recently been reported by another group (62).

**FIGURE 3.**
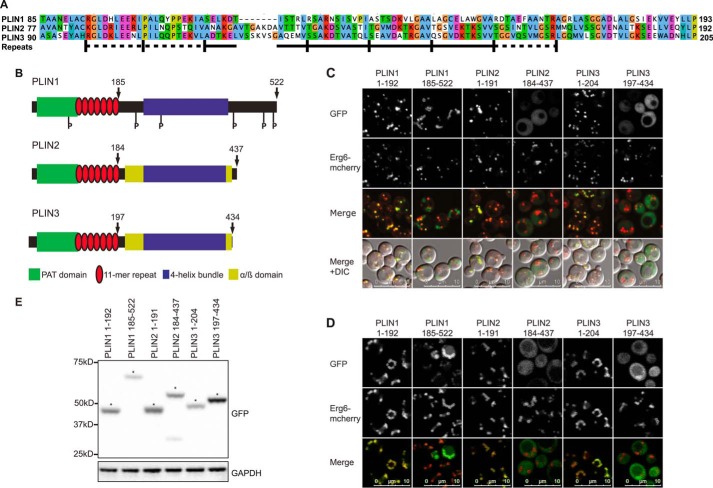
**Amino-terminal 11-mer repeat region is the common element required to target PLINs 1–3 to lipid droplets in *S. cerevisiae*.** *A,* sequence alignment of human PLINs with bioinformatically predicted 11-mer repeats in PLIN1 indicated below. Aligned with PROMALS (76), the repeats were estimated with HHrepID (77) under stringent and relaxed criteria, *p* < 10^−3^ and *p* < 10^−2^ (*full* and *dashed lines,* respectively), and UniProt sequence identifiers O60240, Q99541, O60664. *B,* schematic estimate of the predicted domain architecture of human PLINs 1–3. *Numbers* given are for amino acids. At the amino terminus, these proteins share a highly conserved PAT domain (named in accordance with the original terms used for PLINs 1–3, namely Perilipin, ADRP, and TIP47) and an 11-mer repeat region predicted to form AHs. X-ray crystallography has shown that the carboxyl terminus of PLIN3 folds into a 4-helix bundle and an α/β-domain. Based on sequence similarity, it is predicted that the carboxyl-terminal region of PLIN1 and PLIN2 form similar structures. *C* and *D,* ERG6-mCherry cells expressing the predicted amino- and carboxyl-terminal domains of PLINs 1–3 tagged with GFP at the carboxyl terminus were grown in selective media to log (*C*) or post-diauxic shift (*D*) phase and imaged by confocal microscopy. The brightness and contrast of images were adjusted to aid visualization. Images shown are representative of multiple images acquired in at least four separate experiments except that of PLIN3-(197–434), which was acquired in two separate experiments. *E,* immunoblotting of cells described in *C*. GAPDH was used as a loading control. The *asterisks* highlight the expected size for each protein.

**FIGURE 4.**
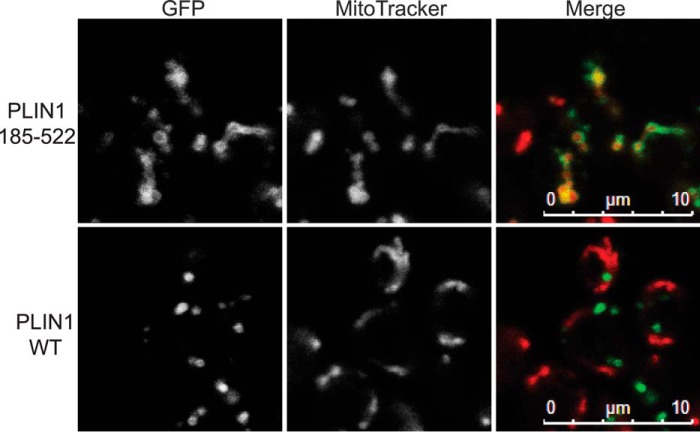
**Carboxyl-terminal domain of PLIN1 localizes to mitochondria in *S. cerevisiae* in log phase.** *S. cerevisiae* expressing PLIN1 WT or amino acids 185–522 tagged with GFP at the carboxyl terminus were grown in selective media to log phase, stained with MitoTracker Orange, and imaged by confocal microscopy. The brightness and contrast of images were adjusted to aid visualization. PLIN1-(185–522) colocalized with MitoTracker labeled mitochondria (*red*), whereas there was no localization of the WT protein to mitochondria. Images shown are representative of multiple images acquired in one experiment with MitoTracker. The study was also performed with a second independent mitochondrial matrix marker (mtDsRed) (78) on more than two separate occasions.

Next, we divided each of PLINs 1–3 in such a way as to separate the predicted amino-terminal 11-mer repeats (Fig. 3*A*) from the carboxyl-terminal 4-helix bundle domain (Fig. 3*B*). LD targeting was preserved in the 11-mer repeat containing regions of PLINs 1–3, whereas the “4-helix bundle” fragments were either diffusely cytosolic in the case of PLINs 2 and 3 (Fig. 3, *C–E*) or localized to the mitochondrial network in the case of PLIN1 (Fig. 4). Interestingly, when studied in the post-diauxic shift phase, which enhances LD growth, the 4-helix bundle region of PLIN1 was targeted to LDs (Fig. 3*D*).

**FIGURE 5.**
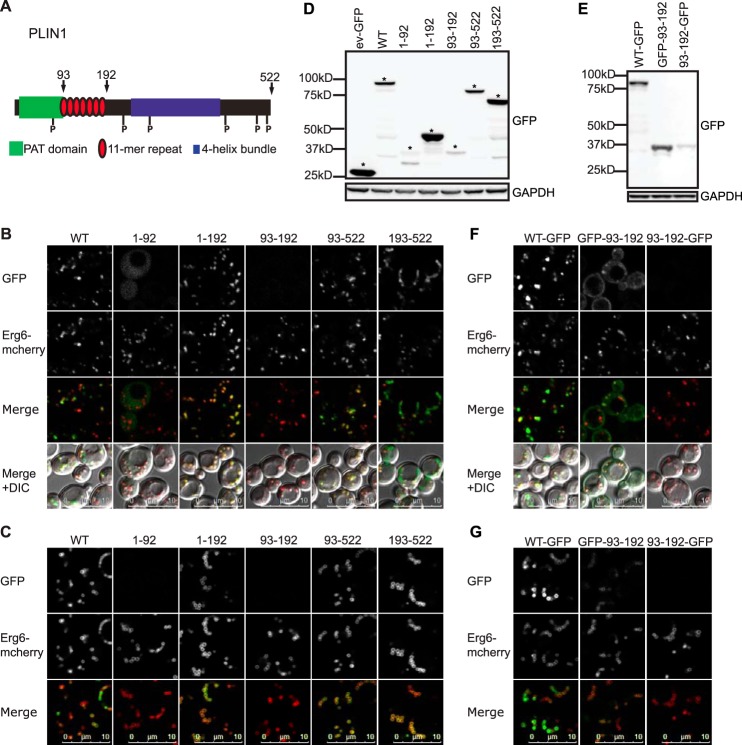
**Localization of PLIN1 deletion fragments to lipid droplets in *S. cerevisiae*.** *A,* schematic estimate of the predicted domain architecture of human PLIN1, highlighting the boundaries of the predicted 11-mer repeat region. Numbers given are for amino acids. Fragments 1–92, 1–192, 93–522, 93–192, and 193–522 were designed to investigate the potential role of the 11-mer repeat region in LD targeting. *B* and *C,* ERG6-mCherry cells expressing PLIN1 fragments tagged with GFP at the carboxyl terminus were grown in selective media to log (*B*) or post-diauxic shift (*C*) phase and imaged by confocal microscopy. The brightness and contrast of images were adjusted to aid visualization. Fragment 93–522 consistently targeted LDs, although fragment 193–522 predominantly targeted LDs in post-diauxic shift phase cells. The predicted 11-mer repeat region (93–192) was poorly expressed, and LD localization was not observed. Images shown are representative of multiple images acquired in four separate experiments except that of 1–92, which was acquired in two separate experiments. *D,* GFP immunoblotting of cells described in *B*. The expected size band for each protein is indicated by the *asterisk*. GAPDH was used as a loading control. *E,* GFP immunoblotting of lysate prepared from ERG6-mCherry cells expressing the indicated GFP tagged PLIN1 fragments and grown to log phase in selective media. Note that PLIN1-(93–192) was more highly expressed when tagged at the amino terminus. GAPDH was used as a loading control. *F* and *G,* ERG6-mCherry cells expressing the indicated GFP tagged PLIN1 fragments were grown in selective media to log (*F*) or post-diauxic shift (*G*) phase and imaged by confocal microscopy. The brightness and contrast of images were adjusted to aid visualization. Some LD targeting was observed for PLIN1-(93–192) when tagged with GFP at the amino terminus. Images shown are representative of multiple images acquired in three (*F*) or two (*G*) separate experiments. *ev,* empty vector.

**FIGURE 6.**
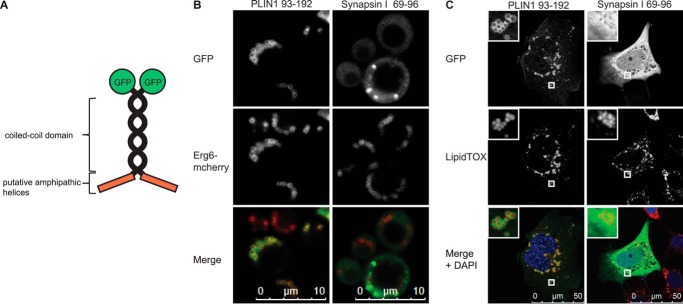
**PLIN1-(93–192) are sufficient to target lipid droplets when expressed in a GFP-tagged bioprobe.** *A,* schematic diagram of the bioprobe consisting of the Golgi microtubule-associated protein coiled-coil domain separating the putative AHs and the GFP tag. *B, S. cerevisiae* ERG6-mCherry cells expressing the indicated peptide-containing bioprobes were grown in selective media to post-diauxic shift phase and imaged by confocal microscopy. The brightness and contrast of images were adjusted to aid visualization. The synapsin I peptide (amino acids 69–96) did not direct the GFP-tagged bioprobe to LDs, whereas the PLIN1-(93–192) was sufficient for LD localization. Images shown are representative of multiple images acquired in three separate experiments. *C,* COS-7 cells were transiently transfected with the indicated peptide containing bioprobe vectors and subsequently loaded with 400 μm oleic acid. Cells were fixed 48 h post-transfection, and the LDs were stained with HCS LipidTOX Deep Red Neutral Lipid Stain. Nuclei were stained with DAPI during mounting. The brightness and contrast of images were adjusted to aid visualization. The synapsin I peptide (amino acids 69–96) did not direct the GFP-tagged bioprobe to LDs, whereas the PLIN1-(93–192) was sufficient for LD localization. *Inset* shows representative zoomed in images of the indicated LDs. Images shown are representative of multiple images acquired in two separate experiments.

To further refine the region of the amino terminus involved in LD targeting, various PLIN1 deletions were generated (Fig. 5*A*). Although these studies suggested that the predicted 11-mer repeat region between amino acids 93 and 192 is required for LD targeting, when expressed in isolation with a carboxyl terminus GFP tag, this region was weakly expressed and did not localize to LDs (Fig. 5, *B–D*). However, when GFP-tagged at the amino-terminal end, this peptide was more highly expressed (Fig. 5*E*) and did, albeit not exclusively, target LDs (Fig. 5, *F* and *G*). As GFP is a relatively bulky tag for a peptide of this size, we also evaluated its targeting capability when expressed in a vector similar to that previously used to study AH membrane targeting by Pranke *et al.* (52) (Fig. 6*A*). This vector expresses dual copies of the AH and separates the GFP tags from the AH with a spacing coiled-coil region. It enables one to compare the targeting properties of different AHs within the same molecular context imposed by the simple coiled-coil geometry (63). In this case, the 11-mer repeat region (amino acids 93–192) was LD-targeted in *S. cerevisiae* as well as in a mammalian cell line (COS-7), whereas the amphipathic region of synapsin I was not localized to LDs (Fig. 6, *B* and *C*).

**FIGURE 7.**
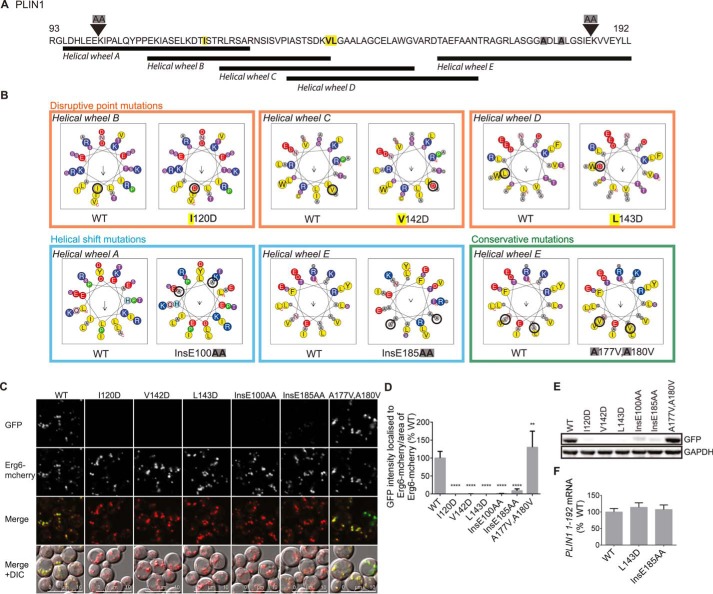
**Mutations designed to disrupt the hydrophobic face of the predicted amphipathic helices decrease the lipid droplet localization of the amino terminus of PLIN1.** *A,* protein sequence of PLIN1-(93–192), with the location of the various mutations introduced. *B,* mutations were designed to disrupt the amphipathic nature of the predicted AHs. Note that the overlapping nature of the predicted helices reflects a degree of uncertainty about exactly where the AHs begin and end. Two classes of disruptive mutations were designed as follows: 1) substitution of a hydrophobic residue with a charged aspartate residue (I120D, V142D, and L143D), and 2) insertion of two alanine residues to produce a register shift in the helices (InsE100AA and InsE185AA). A conservative mutation in which two alanine residues on the hydrophobic face of the helix were replaced by two valine residues (A177V,A180V) was also generated. The effects of these mutations on the helical wheels *A–E* are shown. *C,* ERG6-mCherry cells expressing WT or mutant PLIN1-(1–192) tagged with GFP at the carboxyl terminus were grown to log phase in selective media and imaged by confocal microscopy. The brightness and contrast of images were adjusted to aid visualization. The PLIN1 WT fragment localized to Erg6-mCherry labeled LDs. Introduction of the disruptive mutations (I120D, V142D, L143D, InsE100AA, and InsE185AA) reduced LD localization, whereas the conservative mutation (A177V,A180V) did not disrupt LD targeting. *D,* LD targeting was quantified by calculating the intensity of GFP localized to Erg6-mCherry puncta, normalized to the area of Erg6-mCherry puncta and calculated as a percentage of the mean of the WT from the same experiment. Values are mean ± S.D. of 10–12 different images acquired in two separate experiments. In total, at least 240 cells were quantified per mutant. **, *p* < 0.01; ****, *p* < 0.0001 (one-way ANOVA with Dunnett's multiple comparisons test). *E,* immunoblotting of lysates from cells grown as described in *C*. Disruptive mutations reduced protein expression. GAPDH was used as a loading control. *F, PLIN1-*(*1–192*) mRNA levels from cells grown as described in *C* were quantified by RT-PCR, and expression was normalized to *ACT1* and *PDA1*. There was no difference in mutant mRNA expression compared with WT. Values are mean ± S.D. of three independent biological replicates and calculated as a percentage of the mean of the WT. *DIC*, differential interference contrast.

**FIGURE 8.**
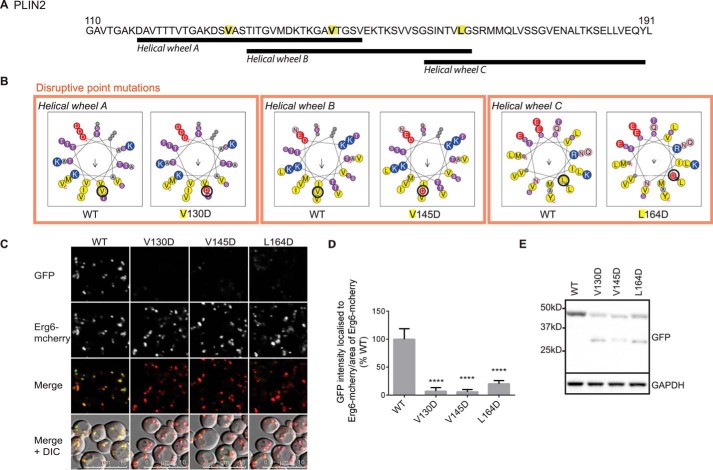
**Mutations designed to disrupt the hydrophobic face of the predicted amphipathic helices decrease the lipid droplet localization of the amino terminus of PLIN2.** *A,* protein sequence of PLIN2 amino acids 110–191, with the location of the various mutations introduced (*yellow*). *B,* mutations were designed to disrupt the amphipathic nature of the predicted AHs by substituting a hydrophobic residue on the hydrophobic face with a charged aspartate residue. The effects of these mutations on the helical wheels *A–C* are shown. *C,* ERG6-mCherry cells expressing WT or mutant PLIN2 amino acids 1–191 tagged with GFP at the carboxyl terminus were grown in selective media to log phase and imaged by confocal microscopy. The brightness and contrast of images were adjusted to aid visualization. WT protein localized to Erg6-mCherry-labeled LDs, whereas the introduction of the disruptive mutations significantly reduced LD targeting. *DIC*, differential interference contrast. *D,* LD targeting was quantified by calculating the intensity of GFP localized to Erg6-mCherry puncta, normalized to the area of Erg6-mCherry, and calculated as a percentage of the mean of the WT from the same experiment. Values are mean ± S.D. of 12 different images acquired in two separate experiments. In total, at least 350 cells per mutant were quantified. ****, *p* < 0.0001 (one-way ANOVA with Dunnett's multiple comparisons test). *E,* GFP immunoblotting of lysates from cells grown as described in *C*. Disruptive mutations reduced protein expression and some degradation is also apparent. GAPDH was used as a loading control.

**FIGURE 9.**
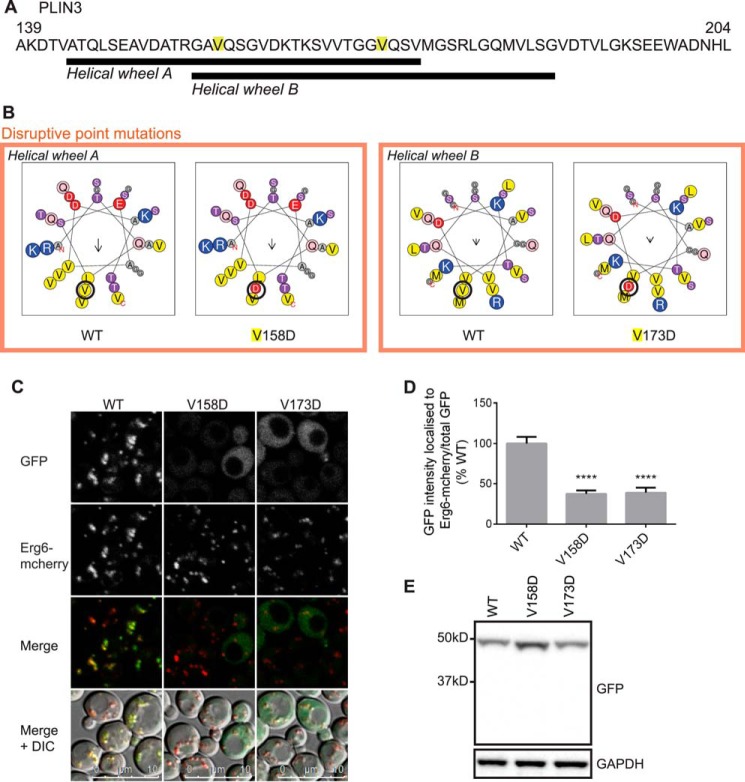
**Mutations designed to disrupt the hydrophobic face of the predicted amphipathic helices decrease lipid droplet localization of the amino terminus of PLIN3.** *A,* protein sequence of PLIN3 amino acids 139–204, with the location of the various mutations introduced (*yellow*). *B,* mutations were designed to disrupt the amphipathic nature of the predicted AHs by substituting a hydrophobic residue on the hydrophobic face with a charged aspartate residue. The effects of these mutations on the helical wheels *A* and *B* are shown. *C,* ERG6-mCherry cells expressing WT or mutant PLIN3 amino acids 1–204 tagged with GFP at the carboxyl terminus were grown to log phase in selective media and imaged by confocal microscopy. The brightness and contrast of images were adjusted to aid visualization. WT protein localized to Erg6-mCherry-labeled LDs. Introduction of the disruptive mutations significantly reduced LD targeting and increased the cytosolic GFP immunofluorescence. *DIC*, differential interference contrast. *D,* LD targeting was quantified by calculating the intensity of GFP localized to Erg6-mCherry puncta, normalized to total GFP, and calculated as a percentage of the mean of the WT from the same experiment. Values are mean ± S.D. of 12 different images acquired in two separate experiments. In total, at least 350 cells per mutant were quantified. ****, *p* < 0.0001 (one-way ANOVA with Dunnett's multiple comparisons test). *E,* GFP immunoblotting of lysates from cells grown as described in *C*. Expression of the mutants is similar to WT. GAPDH was used as a loading control.

Although the 11-mer repeat region is predicted to fold into AHs, this has never been formally investigated. Based on helical wheel predictions, we generated a series of missense and insertion mutations designed to disrupt the predicted AHs in the amino-terminal fragment of *PLIN1*-(1–192), either by inserting charged residues on the hydrophobic face (I120D, V142D, and L143D) or by changing the helix register (InsE100AA and Ins E185AA) (Fig. 7, *A* and *B*). In each case, LD targeting was significantly reduced (Fig. 7, *C* and *D*). In contrast to these disruptive mutations, substituting two alanine residues on the hydrophobic face with larger valine residues (A177V,A180V) resulted in a modest increase in LD targeting (Fig. 7, *A–D*). As has been reported for PLINs 1 and 2 in mammalian cells, mutant proteins that failed to target LDs appeared to be more rapidly degraded as their protein levels were lower than WT and the A177V,A180V mutant despite similar mRNA expression levels (Fig. 7, *E* and *F*). Similarly, mutations designed to disrupt the predicted AHs in the corresponding regions of PLIN2 and 3 significantly reduced LD targeting (Figs. 8 and 9) and protein expression in the case of PLIN2. PLIN3 mutants were more stable in the cytoplasm as reflected by similar protein expression levels and readily discernible cytoplasmic GFP immunofluorescence (Fig. 9*C*).

**FIGURE 10.**
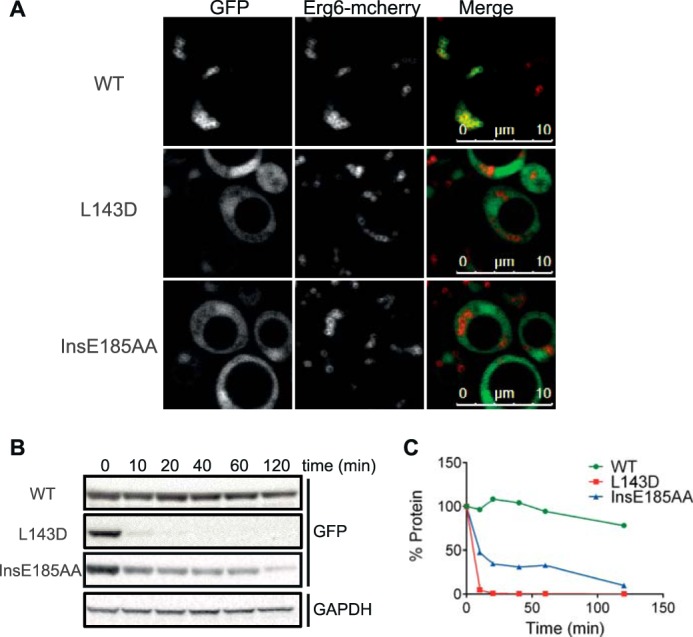
**PLIN1 amino-terminal helix disrupting mutants are more rapidly degraded in *S. cerevisiae*.** *A,* mutations designed to disrupt the hydrophobic face of the predicted AHs prevent the LD localization of PLIN1-(93–192) when expressed in a GFP-tagged bioprobe. Bioprobe schematic is shown in Fig. 6*A*. ERG6-mCherry cells expressing the indicated PLIN1-(93–192) containing bioprobes were grown in selective media to post-diauxic shift phase and imaged by confocal microscopy. The brightness and contrast of images were adjusted to aid visualization. Mutations L143D and InsE185AA prevented LD targeting in all cells viewed, resulting in a cytosolic distribution. Images are representative of multiple images acquired in two separate experiments. *B* and *C,* mutations designed to disrupt the hydrophobic face of the predicted AHs increase the rate of degradation of the amino terminus of PLIN1. ERG6-mCherry cells expressing WT or mutant PLIN1-(1–192) tagged with GFP at the carboxyl terminus were grown in selective media to log phase and treated with cycloheximide. Cell lysate was prepared at the indicated time points. GFP immunoblotting of lysate shows that the mutant proteins not associated with LDs are more rapidly degraded than the WT protein. GAPDH expression is stable across different time points, regardless of which construct was expressed. *B,* immunoblots shown are representative of three separate experiments. *C,* quantification of immunoblots shown in *B*. The protein remaining at each time point was normalized to GAPDH and calculated as a percentage of the 0-min time point. Immunoblot quantification was similar whether normalized to GAPDH or to protein loading.

To address the possibility that the primary reason for reduced LD targeting of disruptive PLIN1 AH mutants was simply that they were not expressed, we first expressed two of these mutants (L143D and InsE185AA) in the CC bioprobe vector described by Pranke *et al.* (52) (see Fig. 6*A*), as this construct is more likely to stabilize expression regardless of LD targeting. This was indeed the case, and both disruptive mutations led to increased cytosolic GFP signal with no apparent LD targeting in any cells imaged (Fig. 10*A*). We hypothesized that mutants, which prevent LD targeting without the Pranke *et al.* (52) CC vector, are more rapidly degraded than proteins associated with the LD. To formally demonstrate this, we assessed protein expression levels of the WT and mutant (L143D and InsE185AA) GFP-tagged PLIN1 amino-terminal fragment (amino acids 1–192) following cycloheximide exposure. These data confirmed that the mutants are more rapidly degraded than WT protein in this context (Fig. 10, *B* and *C*).

**FIGURE 11.**
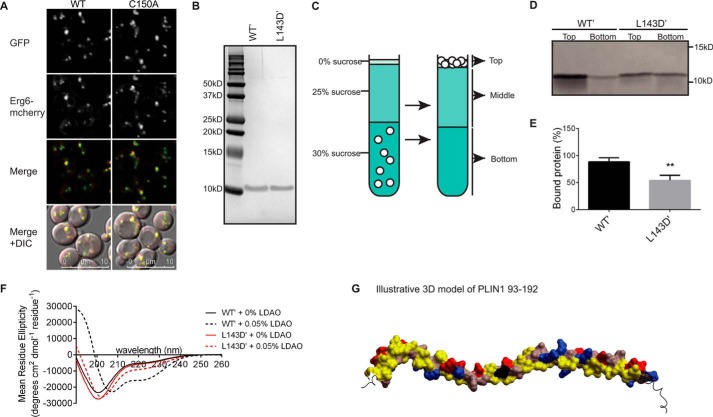
**PLIN1-(93–192) associate with lipid droplets *in vitro* and manifest a substantial increase in helicity in the presence of detergent.** *A,* ERG6-mCherry cells expressing PLIN1-(1–192) WT or C150A tagged with GFP at the carboxyl terminus were grown in selective media to log phase and imaged by confocal microscopy. The brightness and contrast of images were adjusted to aid visualization. The C150A mutant protein localized to LDs to a similar extent as the WT protein. Images shown are representative of multiple images acquired in three separate experiments. *DIC*, differential interference contrast. *B,* Coomassie-stained gel of the indicated purified PLIN1-(93–192) peptides. The prime (′) indicates that purified proteins harbor the C150A mutation to eliminate non-native disulfide bond formation during purification. Equal protein concentrations were resolved as determined by amino acid analysis. *C,* LD flotation diagram. 1,2-Dioleoyl-*sn*-glycero-3-phosphocholine/1,2-dioleoyl-*sn*-glycero-3-phosphoethanolamine (80:20) artificial LDs and protein are incubated together for 30 min, adjusted to 30% sucrose, and loaded at the bottom of a sucrose gradient consisting of 25 and 0%, sucrose respectively. During ultracentrifugation, LDs and bound protein float to the top of the gradient, whereas unbound protein remains in the bottom fraction. *D,* L143D mutation reduces LD association compared with WT PLIN1-(93–192). PLIN1-(93–192) WT′ and L143D′ (both at 0.5 μm) were incubated with artificial LDs and separated on a sucrose gradient. Equal volumes of the resulting fractions were resolved by SDS-PAGE and stained by silver staining. The gel shown is representative of three separate experiments. *E,* quantification of the band intensities indicates that significantly more PLIN1-(93–192) WT′ was detected in the top LD fraction when compared with the L143D′ peptide. Values are mean ± S.D. of three separate experiments. *F,* circular dichroism spectroscopy of purified PLIN1-(93–192) peptides prepared at 10 μm in 10 mm potassium phosphate, pH 7.4, in the presence or absence of 0.05% LDAO. Spectra were collected on an Aviv Model 410 at 25 °C. Note the increase in helicity of the WT′ peptide in the presence of LDAO. A smaller increase in helicity is observed for the L143D′ mutant. Spectra shown are representative of four separate experiments. *G,* illustrative three-dimensional model of PLIN1-(93–192). Color coding of the residue surface is as follows: *red*, charged negative; *blue*, charged positive; *pink*, polar uncharged; *yellow*, hydrophobic; *black*, L143. Amino terminus is on *right*. Built on HHpred (79) sequence alignment of PLIN1-(78–197) and apolipoprotein A-IV (112–229, Protein Data Bank code 3s84) using program Modeler (80) and displayed with BrowserPro (MolSoft L.L.C.).

To reinforce these data, which collectively implicate AHs in LD targeting, we went on to express and purify the 11-mer repeat region of PLIN1, namely amino acids 93–192. After confirming that a PLIN1 C150A mutation did not affect LD targeting of PLIN1-(1–192)-GFP *in vivo* (Fig. 11*A*), this mutation (C150A) was incorporated into the purification clones to eliminate non-native disulfide bridge formation during purification, as indicated by the ′ in the following text. Purified PLIN1-(93–192) WT′ associated with artificial LDs separated on a sucrose gradient (Fig. 11, *B–E*). However, a disruptive mutant (L143D), which we had previously shown reduced LD targeting *in vivo* (Figs. 7 and 10), significantly inhibited this association (Fig. 11, *B–E*). Analysis of the secondary structure of this purified peptide by circular dichroism spectroscopy suggested that both the WT′ and L143D′ mutant peptides were largely unstructured in solution (Fig. 11*F*). The addition of a membrane-mimicking detergent (LDAO) at a concentration above the critical micelle concentration resulted in a substantial increase in the helicity of the WT′ peptide (Fig. 11*F*), whereas the change in helicity of the L143D′ mutant peptide was considerably less than that of the WT′. Deconvolution of the CD spectra estimated that the WT′ peptide increased in helicity from 15 to 59% in the presence of LDAO, whereas the L143D′ mutant peptide had a limited increase in helicity from 16 to 23%. Thus, although the WT′ peptide folds into helices in the presence of detergent micelles, this is significantly disrupted by insertion of a charged amino acid in what would otherwise become part of the helical hydrophobic face (Fig. 11*G*).

## Discussion

Collectively these data establish that the 11-mer repeat regions, which lie between the amino-terminal PAT domain (Fig. 3*B*), involved in interacting with HSL, and the 4-helix bundle domain, are sufficient to mediate LD targeting. The precise boundaries of the 11-mer repeat region are difficult to predict with absolute certainty on the basis of sequence analysis alone and have to be experimentally evaluated. Our predictions were influenced by the notion that the PAT domain has been shown to interact with HSL and is roughly located in the first 100 amino acids of PLINs 1–3. We estimated the carboxyl-terminal limit of this region based on the known crystal structure of the 4-helix bundle domain of PLIN3 (47). We acknowledge that our “fragments” may not be the precise limits of the 11-mer repeat region. Furthermore, these regions, at least when purified, are largely unstructured in solution but fold into helices in the presence of detergent micelles. The idea that this region is unstructured when the proteins are not associated with membranes is consistent with CD data indicating that the amino terminus (consisting of the PAT domain and 11-mer repeat region) of soluble full-length PLIN3 has relatively little helical content (46). Importantly, mutations designed to disrupt the hydrophobic face of these helices significantly impair LD targeting and, interestingly, one of these mutants significantly reduces helical folding in the presence of detergent micelles. The latter data are consistent with helical folding being significantly reduced by loss of the amphipathicity of a key helix.

Our current understanding of these data is that the 11-mer repeat region behaves as a typical “nascent helix.” This concept was introduced by Dyson *et al.* in 1988 (64) and has since been widely invoked. The idea is that the 11-mer repeat region does not have a rigid well defined structure. Instead, it is an ensemble of rapidly interconverting helical loops and random coil structure in equilibrium. The helical propensity is strongly indicated by secondary structure prediction but only becomes observable if the helical conformation is stabilized by external forces. This could be a helix-promoting milieu such as micelles or interaction with a suitable membrane environment. The external surface serves as a nucleation point for the whole conformation, so a single amino acid mutation can weaken the nucleation and shift the equilibrium toward the unfolded “nascent” conformation. This concept has been previously considered by Antonny and co-workers (65) in the context of AH association with bilayer membranes.

At least, when expressed in *S. cerevisiae*, the amino-terminal fragments (PLIN1-(1–192)-GFP and PLIN2-(1–191)-GFP) are stabilized by association with the LD surface. In contrast, mutations that reduce LD targeting in the PLIN3-(1–204)-GFP construct do not appear to reduce protein expression. These findings are broadly consistent with the behavior of endogenous PLINs 1–3, as we and others have reported (14, 21–28). At least in the case of PLIN1, this tendency for the protein to be degraded when not associated with a LD prevents sequestration of ABHD5 by PLIN1 in a cytosolic or alternative context, which might impair its subsequent ability to co-activate ATGL on the LD surface in response to a lipolytic stimulus. These data could also explain the apparently more dramatic reductions in *in vivo* LD targeting of the AH mutants (Fig. 7) compared with the ∼50% difference observed in *in vitro* LD association of the purified PLIN1-(93–192) peptide (Fig. 11), as the latter would not be degraded in an *in vitro* system. Our collective interpretation of these data is that the mutations designed to disrupt AH formation significantly reduce LD association, and in an *in vivo* context, this facilitates accelerated degradation of the protein leading ultimately to a marked reduction in expression of these mutants.

Secondary structure prediction and homology with proteins such as apolipoproteins and α-synuclein (49) known to fold into AHs indicate that the 11-mer repeat region is likely to fold into a long α-helix interrupted by irregularities and kinks. A tentative three-dimensional model (Fig. 11*G*) illustrates the formation of a hydrophobic face twisting along the axis of the helix. The mutation L143D substantially decreases the helix-forming propensity (Fig. 11*F*) of the peptide, “breaks” the hydrophobic face, and abolishes LD targeting *in vivo*. Leu-143 thus appears to be a key component of the LD surface detection sequence. Although the three-dimensional model in Fig. 11*G* suggests a continuous helix, in reality the boundaries of the 11-mer repeat region remain uncertain, and it is very likely that the entirety of PLIN1-(93–192) does not all fold into helices.

The 11-mer repeats are highly conserved, being readily identifiable even in the simplest known PLIN orthologues (*Trichoplax*, *Dictyostelium*, fungi, and *Drosophila*). The 4-helix bundle domain is more variable (47) or even absent in PLINs of some lower organisms. Nevertheless, some studies have reported that this region is also capable of membrane targeting (34–36, 40, 43, 45), and we too observed that, at least in the case of PLIN1, this region did localize to LDs in the post-diauxic shift phase. Helix bundles in apolipoproteins are known to be able to unfold and associate with phospholipid membranes (66). Detailed homology analysis (14, 47) suggests that the corresponding region of PLIN1 would form a helix bundle, albeit with somewhat irregular helices incorporating some proline residues. The latter may increase the flexibility of the helical regions facilitating interaction with the LD surface, as has been proposed for apolipoproteins (49).

Interestingly, we have previously shown that the β-sheets, known to zip the 4-helix bundle together in PLIN3, are unlikely to exist in PLIN1 due to the insertion of an extra exon at some point in the evolution of this isoform (14). Thus, we hypothesize that the 4-helix bundle of PLIN1 is less stable than that of PLIN3 and might find its minimal conformational energy by unfolding and associating with a membrane. This could account for our own observations that the carboxyl-terminal region of PLIN1 can associate with larger yeast LDs in the post-diauxic shift phase. Subramanian and co-workers (34, 35) have proposed a variation on this model in which they suggest that three short hydrophobic sequences within this region are involved in LD targeting, although they acknowledge that they have yet to formally provide a mechanistic basis for their proposal.

Although the tendency is to assume that the phospholipid monolayer surrounding the neutral lipid core of an LD is oriented and manifests properties similar to half of a phospholipid bilayer, this may not be the case (50). So exactly what the equivalent of a membrane “packing defect” would be on a LD is uncertain. Nevertheless, the fact that the unstructured 11-mer repeat region of PLIN1 is able to fold into helices in the presence of detergent micelles implies that, *in vivo*, this region recognizes and folds into some form of hydrophobic surface “defect.” As we and others have reported (11, 14, 41, 43, 67–74), PLINs appear to compete for sites on the LD surface, implying that these packing defects are limited. Presumably, specific properties of the AHs of each of the PLINs are a key factor in determining the outcome of this competitive LD association.

In conclusion, these data establish that the initially unstructured 11-mer repeat regions of PLINs 1–3 can facilitate the association of PLINs with the surface of LDs. We hypothesize that initial detection of, and interaction with, the LD surface by the 11-mer repeats triggers the 4-helix bundle to unfold and anchor the protein to the LD, although we acknowledge that this proposed sequential model has yet to be formally proven. In the case of PLIN1, we suggest that secondary unfolding of the 4-helix bundle occurs at a lower energetic threshold than in PLIN3, where the 4-helix bundle is stabilized by the α/β-domain, although this too will require further experimental confirmation. These data highlight the importance of AHs in detecting specific phospholipid membrane environments (75), which in the case of PLINs means that they are ideally placed to precisely coordinate lipid release from droplets.

## Author Contributions

D. B. S. conceived, coordinated the study, and wrote the manuscript. E. R. R. designed and performed the experiments, prepared the figures, and contributed to the manuscript. M. L. M. designed and performed the protein expression and purification and contributed to the manuscript. A. H. performed experiments. I. I. assisted E. R. R. with cloning. A. D. B. and S. S. provided invaluable support and advice for work in *S. cerevisiae*. M. M. O. and A. R. T. provided guidance for artificial LD preparation, and M. M. O. also advised with CD experiments. V. S. performed bioinformatics analyses and contributed to the manuscript. S. P. provided general advice and guidance. All the authors reviewed and edited the manuscript.

## Footnotes

2 The abbreviations used are:

LD: lipid droplet
PLIN: perilipin
ATGL: adipose triglyceride lipase
HSL: hormone-sensitive lipase
AH: amphipathic helix
CC: coiled-coil
LDAO: *N,N*-dimethyldodecylamine *N*-oxide
ANOVA: analysis of variance.
