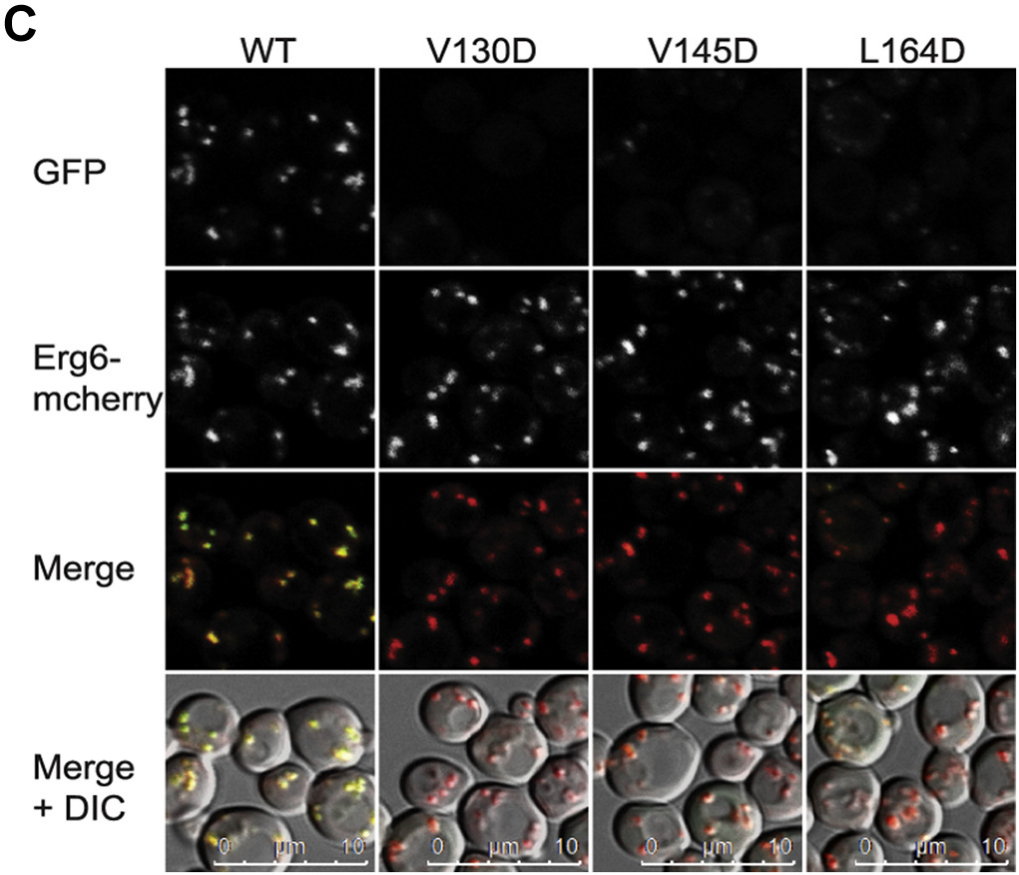# Correction: Conserved amphipathic helices mediate lipid droplet targeting of perilipins 1–3

**DOI:** 10.1016/j.jbc.2021.101490

**Published:** 2021-12-20

**Authors:** Emily R. Rowe, Michael L. Mimmack, Antonio D. Barbosa, Afreen Haider, Iona Isaac, Myriam M. Ouberai, Abdou Rachid Thiam, Satish Patel, Vladimir Saudek, Symeon Siniossoglou, David B. Savage

In Figure 8*C*, the L130D Merge + DIC image was inadvertently duplicated and presented as both the L130D Merge + DIC image and the L164D Merge + DIC image. The L164D Merge + DIC image has therefore been replaced with the correct original source image. This correction does not affect the quantification in Figure 8*D*, the results or conclusions of the experimental work.

**Figure:**